# Supplementary material for: The Systems Biology Research Tool: evolvable open-source software
Source: BMC Syst Biol. 2008 Jun 29;2:55. doi: 10.1186/1752-0509-2-55 (PMC2446383; doi:10.1186/1752-0509-2-55)
Supplement: Additional file 1 — SBRT Archive. An archive of the current version of the Systems Biology Research Tool. [file 1752-0509-2-55-S1.zip › sbrt-1.4.0/doc/developers_guide/api/sbrt/kernel/fba/class-use/FluxConstraints.html]

Uses of Class sbrt.kernel.fba.FluxConstraints


|  |  |  |
| --- | --- | --- |
| **Overview** \| **Package** \| **Class** \| **Use** \| **Tree** \| **Deprecated** \| **Index** \| **Help** | |  |
| PREV   NEXT | **FRAMES**    **NO FRAMES**     **All Classes** |  |


---


## **Uses of Class sbrt.kernel.fba.FluxConstraints**

| Packages that use FluxConstraints | |
| --- | --- |
| **sbrt.kernel.fba** | Provides classes and interfaces for creating objects relevant to Flux Balance Analysis. |
| **sbrt.kernel.fba.proc** | Provides classes and interfaces for executing Flux Balance Analysis processes. |
| **sbrt.shell.io.fba** | Provides classes and interfaces for reading and writing files relevant to Flux Balance Analysis. |
| **sbrt.shell.mng.fba** | Provides classes and interfaces for creating Flux Balance Analysis process managers. |

Uses of FluxConstraints in sbrt.kernel.fba

| Methods in sbrt.kernel.fba that return FluxConstraints | |
| --- | --- |
| `FluxConstraints` | `Fluxome.getConstraints()`             Returns the set of all constraints for fluxes and mathematical expressions of fluxes in this fluxome. |
| `FluxConstraints` | `IrrevRxnFluxomeV1.getConstraints()`             Returns all constraints in this fluxome. |

| Methods in sbrt.kernel.fba with parameters of type FluxConstraints | |
| --- | --- |
| `IrrevRxnFluxome` | `IrrevRxnFluxome.replaceConstraints(FluxConstraints constraints, double constraintTolerance)`             Returns a copy of this fluxome, but with all constraints replaced by those provided. |
| `Fluxome` | `Fluxome.replaceConstraints(FluxConstraints constraints, double constraintTolerance)`             Returns a copy of this fluxome, but with all constraints replaced by those provided. |
| `IrrevRxnFluxomeV1` | `IrrevRxnFluxomeV1.replaceConstraints(FluxConstraints fluxConstraints, double constraintTolerance)`             Returns a copy of this fluxome, but with all constraints replaced by those provided. |
| `CatalyzedFluxome` | `CatalyzedFluxome.replaceConstraints(FluxConstraints constraints, double constraintTolerance)`             Returns a copy of this fluxome, but with all constraints replaced by those provided. |

Uses of FluxConstraints in sbrt.kernel.fba.proc

| Methods in sbrt.kernel.fba.proc with parameters of type FluxConstraints | |
| --- | --- |
| `static void` | `CvRxnDeletionProcess.checkForConflict(FluxConstraints fc, java.util.Set<java.lang.String> deletionNames)`             Ensures that the provided flux constraints and set of reaction names do not conflict. |
| `static void` | `CvFbaOptProcess.restoreConstraints(FluxConstraints constraints, FluxCvComputer computer)`             Restores the provided constraints in the provided constraint-variation computer. |
| `static void` | `CvFbaOptProcess.setConstraints(FluxConstraints constraints, FluxCvComputer computer)`             Sets the provided constraints in the provided constraint-variation computer. |

| Method parameters in sbrt.kernel.fba.proc with type arguments of type FluxConstraints | |
| --- | --- |
| `void` | `CvFbaOptimizationExperiment.setConstraintSource(java.lang.Iterable<FluxConstraints> constraintSource)`             Sets the constraint variation input source. |
| `void` | `CvRxnDeletionProcess.setConstraintSource(java.lang.Iterable<FluxConstraints> constraintSource)`             Sets the constraint variation input source. |
| `void` | `SvInitialPointProcess.setConstraintSource(java.lang.Iterable<FluxConstraints> constraintSource)`             Sets the constraint variation input source. |
| `void` | `CvObjectiveFunctionProcess.setConstraintSource(java.lang.Iterable<FluxConstraints> constraintSource)`             Sets the constraint variation input source. |
| `void` | `CvFluxIntervalProcess.setConstraintSource(java.lang.Iterable<FluxConstraints> constraintSource)`             Sets the constraint variation input source. |
| `void` | `SvCdHarFluxVectorProcess.setConstraintSource(java.lang.Iterable<FluxConstraints> constraintSource)`             Sets the constraint variation input source. |
| `void` | `CvCatalystDeletionProcess.setConstraintSource(java.lang.Iterable<FluxConstraints> constraintSource)`             Sets the constraint variation input source. |

Uses of FluxConstraints in sbrt.shell.io.fba

| Methods in sbrt.shell.io.fba that return FluxConstraints | |
| --- | --- |
| `static FluxConstraints` | `FluxIntervalInputFile.convertConstraints(java.util.Map<LinearComb<java.lang.String>,Interval> intervals)`             Converts the provided intervals into a `FluxConstraints` object. |

| Methods in sbrt.shell.io.fba that return types with arguments of type FluxConstraints | |
| --- | --- |
| `java.util.Iterator<FluxConstraints>` | `FluxIntervalInputFile.iterator()`             Returns an iterator over the flux intervals contained in this file. |

Uses of FluxConstraints in sbrt.shell.mng.fba

| Methods in sbrt.shell.mng.fba that return FluxConstraints | |
| --- | --- |
| `static FluxConstraints` | `FbaOptManager.getConstraints(ManagerInput<java.lang.String,java.lang.String> input, java.io.PrintWriter out, Fluxome fluxome)`             Opens the file denoted by `FbaOptManager.CONSTRAINTS_FILE` in the provided manager input and returns the set of flux constraints it contains. |
| `static FluxConstraints` | `RandomFluxVectorManager.getConstraints(ManagerInput<java.lang.String,java.lang.String> input, java.io.PrintWriter out, Fluxome fluxome)`             Opens the file denoted by `FbaOptManager.CONSTRAINTS_FILE` in the provided manager input and returns the set of flux constraints it contains. |
| `static FluxConstraints` | `FbaOptManager.getConstraints(java.lang.String fileName, Fluxome fluxome)`             Returns the set of constraints contained in the file with the provided name. |
| `static FluxConstraints` | `RandomFluxVectorManager.getConstraints(java.lang.String fileName, Fluxome fluxome)`             Returns the set of constraints contained in the file with the provided name. |
| `static FluxConstraints` | `InitialPointManager.getIntervals(ManagerInput<java.lang.String,java.lang.String> input, java.io.PrintWriter out, Fluxome fluxome)`             Opens the file denoted by `InitialPointManager.FLUX_INTERVAL_FILE` in the provided manager input and returns the set of flux intervals it contains. |
| `static FluxConstraints` | `InitialPointManager.getIntervals(java.lang.String fileName, Fluxome fluxome)`             Returns the set of flux intervals contained in the file with the provided name. |

---


|  |  |  |
| --- | --- | --- |
| **Overview** \| **Package** \| **Class** \| **Use** \| **Tree** \| **Deprecated** \| **Index** \| **Help** | |  |
| PREV   NEXT | **FRAMES**    **NO FRAMES**     **All Classes** |  |


---
